# In Vitro Characterization of Insulin-Loaded Soft Contact Lenses and Their Effect on Corneal Epithelial Cell Viability and Permeability

**DOI:** 10.3390/pharmaceutics18070779

**Published:** 2026-06-25

**Authors:** Maria Romaguera, Maria Vivero-Lopez, Affiong Iyire, Raquel Gil-Cazorla, Francisco Arnalich-Montiel, Gonzalo Bernabeu, Gonzalo Carracedo

**Affiliations:** 1 Ocupharm Research Group, Department of Optometry and Vision, Faculty of Optics and Optometry, Complutense University of Madrid, 28037 Madrid, Spain; mromague@ucm.es; 2 C035—R+D in Drug Dosage Forms and Drug Delivery Systems, Health Research Institute of Santiago de Compostela (IDIS), 15706 Santiago de Compostela, Spain; mariavivero.lopez@usc.es; 3 Aston Pharmacy School, College of Medicine Pharmacy & Biosciences, Aston University, Birmingham B4 7ET, UK; a.iyire@aston.ac.uk; 4 Optometry and Vision Science Research Group (OVSRG), School of Optometry, Aston University, Birmingham B4 7ET, UK; r.gil-cazorla@aston.ac.uk; 5 Department of Ophthalmology, Ramón y Cajal University Hospital, 28034 Madrid, Spain; francisco.arnalich@salud.madrid.org; 6 Instituto Ramón y Cajal de Investigación Sanitaria (IRYCIS), 28034 Madrid, Spain; 7 HM Eye Center, 28015 Madrid, Spain; gonbernabeu@gmail.com

**Keywords:** insulin, soft contact lenses, drug-eluting contact lenses, ocular drug delivery, corneal wound healing, corneal ulcer

## Abstract

**Background/Objectives**: Corneal epithelial defects and ulcers remain a significant clinical challenge, often leading to vision impairment and requiring prolonged treatment. In this context, topical insulin has recently gained attention in ophthalmic research. However, conventional eye drops suffer from short residence time and poor bioavailability. To overcome these limitations, the present study evaluates, for the first time in vitro, multiple commercially available soft contact lenses as sustained insulin delivery platforms, analyzing how protein loading influences the essential physicochemical and optical properties of these materials. **Methods**: The physicochemical properties of eight different commercially available soft contact lens materials, including light transmittance, wettability, and central thickness, were examined before and after insulin loading via a soaking method. Loading efficiency and in vitro release profiles were assessed over time. Corneal cytotoxicity and permeability were evaluated using a human epithelial cell-based model (HCE-2). **Results**: Among the eight commercial materials screened, Nesofilcon A, Stenfilcon A, and Delefilcon A were selected due to their superior physicochemical performance after insulin loading. At initial concentrations of 1750 and 875 μg/mL, drug loading efficiency reached maximum values of up to 69.3% and 63.1%, with cumulative release values reaching up to 32.4% and 55.1% after 24 h, respectively. Permeability studies confirmed effective insulin diffusion across the HCE-2 cell layer, while cell viability assays indicated no significant cytotoxicity at the lower loading concentration. **Conclusions**: Insulin-loaded commercial soft contact lenses represent a promising drug–device combination product for the management of persistent epithelial defects and refractory corneal ulcers. These in vitro findings suggest that this approach may enhance drug performance by prolonging residence time and improving corneal bioavailability, while maintaining essential lens properties. However, further in vivo and clinical studies are required to confirm these potential benefits and establish therapeutic efficacy for the management of persistent epithelial defects.

## 1. Introduction

Corneal ulcers are defects of the corneal surface involving damage to the epithelial layer and underlying stroma and represent a significant ocular health concern worldwide, contributing to substantial morbidity and potential vision loss [1]. Annually, between 30,000 and 75,000 cases of corneal ulcers are reported in the United States [2]. These ulcers arise primarily from infectious origins, with bacteria such as *Staphylococcus aureus*, coagulase-negative staphylococci, and *Pseudomonas aeruginosa* being the most commonly implicated pathogens [1]. While viral infections, particularly herpes simplex virus, fungal organisms including *Aspergillus* and *Fusarium* species, and parasitic protozoa like *Acanthamoeba* are significant causes of keratitis, their role in established corneal ulcers is less prominent but clinically relevant in certain settings [1]. Additionally, non-infectious factors, including trauma, prolonged contact lens use, ocular surface diseases like dry eye, and systemic conditions such as diabetes and immunosuppression, can also predispose the cornea to ulceration by compromising epithelial integrity and immune defense [1]. These diverse etiologies contribute to the clinical complexity and variable progression of corneal ulcers, making timely diagnosis and treatment critical to prevent vision loss.

In case of infectious corneal ulcers, initial treatment typically involves empirical antimicrobial therapy tailored to the most likely causative pathogen, supported by culture and sensitivity testing [1,3]. Adjunctive therapies may include topical corticosteroids to control inflammation, cycloplegics for pain relief, and measures to promote epithelial healing, such as lubricants and bandage contact lenses [4,5].

Non-infectious corneal ulcers represent some of the most complex and challenging corneal conditions to manage effectively [6]. Clinical examination remains the primary tool for assessing corneal injuries, with diagnostic agents such as fluorescein aiding evaluation [7]. A range of cycloplegic and anti-inflammatory medications, including commonly used steroids and non-steroidal agents, and off-label oral drugs, help manage inflammation and pain while balancing side effects [7]. Additionally, amniotic membrane therapies, blood-derived treatments, and temporary protective methods like bandage contact lenses and tarsorrhaphy play an increasingly important role in promoting corneal healing [7]. However, despite these therapeutic options, effective regeneration of the corneal epithelium remains limited in many cases. Consequently, there is growing interest in novel regenerative and neurotrophic agents, such as recombinant human nerve growth factor and topical insulin, which have recently emerged or are in late-stage clinical trials, attracting substantial interest from researchers and clinicians for the treatment of these injures [7].

Particularly, insulin is gaining attention as a potential agent to enhance corneal wound repair by promoting epithelial cell proliferation and migration, thereby contributing to more rapid closure of the epithelial defects [8]. Insulin is a peptide hormone produced and secreted by pancreatic beta cells in the islets of Langerhans [9]. It plays a crucial role in regulating blood glucose levels by promoting glucose uptake into tissues such as skeletal muscle, liver, and adipose tissue [9]. Beyond its role in glucose homeostasis, insulin influences several other cellular processes, including glycogen synthesis, protein and lipid metabolism, amino acid transport, and the modulation of DNA synthesis and gene expression via signaling pathways [10]. From a therapeutic standpoint, insulin is primarily administered for the management of type 1 diabetes, advanced or decompensated type 2 diabetes, gestational diabetes, and acute hyperglycemic emergencies such as diabetic ketoacidosis and hyperosmolar hyperglycemic states [11]. However, its therapeutic relevance extends further, as topical insulin has been shown to significantly improve tissue repair and wound healing by modulating inflammatory responses, promoting cell proliferation, and enhancing collagen production [12,13,14]. Additionally, insulin stimulates angiogenesis, supplying the wound area with oxygen and nutrients necessary for tissue repair [15].

In ophthalmology, topical insulin has attracted growing attention due to its potential to promote corneal wound healing, particularly in conditions such as persistent epithelial defects and neurotrophic ulcers [16]. Experimental studies in diabetic animal models, together with clinical evidence, indicate that insulin eye drops can accelerate epithelial regeneration, attenuate inflammatory processes, and support corneal nerve recovery. These effects are mediated through several molecular pathways, including the modulation of neuropeptides such as substance P and calcitonin gene-related peptide, as well as activation of insulin/IGF-1 receptor signaling. In particular, engagement of these receptors triggers downstream PI3K/Akt/mTOR pathway, which plays a central role in regulating corneal epithelial cell migration, proliferation, and metabolism, all of which are essential processes for corneal wound healing [16,17,18]. Despite these promising findings, the clinical use of conventional insulin eye drops is limited by rapid elimination from the ocular surface and low bioavailability, which requires frequent administration and may compromise treatment adherence and overall effectiveness [16,19].

To address these challenges, the use of contact lenses as ocular drug delivery systems has emerged as a promising strategy [20]. By maintaining prolonged contact with the corneal surface, drug-eluting lenses can significantly increase the residence time of drugs, thereby enhancing their bioavailability up to 50% compared with standard eye drop formulations [21]. In addition, these platforms enable controlled and sustained release at the site of action, improving therapeutic outcomes while reducing systemic exposure, dosing frequency, and discomfort associated with repeated instillation or invasive procedures [20]. However, despite the growing interest in both topical insulin and contact lens-based drug delivery systems, there is still a lack of systematic evaluation of commercially available soft contact lenses as insulin delivery platforms, particularly in terms of drug release, epithelial permeation, and cytocompatibility [22]. Commercially available soft contact lenses (SCLs) comprise a wide range of materials, including conventional hydrogels (CH) and different generations of silicone hydrogels (SH) [23]. These materials vary in key physicochemical properties such as water content, oxygen permeability, surface wettability, and light transmittance. Such characteristics play a crucial role in determining the capacity of the lenses to load, retain, and release insulin effectively, while maintaining optical transparency and wearer comfort [23].

This study aimed to evaluate the potential of commercially available SCLs as platforms for ocular insulin delivery, including the influence of physicochemical material properties on drug loading and release, as well as their ability to enable insulin permeation across the corneal epithelium while maintaining cytocompatibility. To achieve this, a range of eight different commercial conventional and silicone-based soft contact lenses was loaded with insulin and subjected to comprehensive characterization. Physical and optical parameters, including water content, light transmittance, wettability, refractive index, central thickness, and power profile, were assessed before and after loading to confirm that insulin loading did not compromise key functional properties of the lenses. Based on these results, three materials were selected for further in vitro evaluation. Insulin loading capacity and release kinetics were then investigated to determine the ability of each lens material to incorporate and deliver insulin effectively. In addition, in vitro cell viability and permeability studies were conducted to assess the safety of the drug-loaded lenses and insulin permeability across an ocular epithelial model. To our knowledge, this is the first study to systematically investigate insulin loading and release from different commercially available SCL materials, together with evaluation of epithelial permeation and cytocompatibility using a human corneal epithelial cell model.

## 2. Materials and Methods

### 2.1. Materials

Six commercially available silicone hydrogel SCL materials were evaluated in this study: Balafilcon A (PureVision^®^, Bausch & Lomb, Inc., Jacksonville, FL, USA), Stenfilcon A (MyDay^®^, CooperVision, Inc., Fairport, NY, USA), Comfilcon A (Biofinity^®^, CooperVision, Inc., Fairport, NY, USA), Delefilcon A (Dailies Total1^TM^, Alcon Inc., Fort Worth, TX, USA), Lehfilcon A (Total30^TM^, Alcon Inc., Fort Worth, TX, USA) and Lotrafilcon A (Air Optix^TM^ Night & Day^TM^, Alcon Inc., Fort Worth, TX, USA). Two conventional hydrogel SCL materials were also included: Nesofilcon A (Biotrue^®^, Bausch & Lomb, Bridgewater, NJ, USA) and Etafilcon A (Acuvue^®^ Moist, Johnson & Johnson Vision Care, Inc., Jacksonville, FL, USA). Table 1 summarizes the manufacturer-reported properties of these SCLs. All lenses were obtained from the manufacturers in their original packaging, and in three dioptric powers (−6.00, −0.50, +6.00 D).

Human insulin (Actrapid^®^, 100 IU/mL; Novo Nordisk, Bagsværd, Denmark) diluted in 0.4% polyethylene glycol (PEG) 400–0.3% propylene glycol eye drops (Systane^®^ Ultra, Alcon Inc., Fort Worth, TX, USA) was used for lens loading. Phosphate-buffered saline (PBS 1×, pH 7.4) was freshly prepared in the laboratory using analytical-grade NaCl, KCl, KH_2_PO_4_, Na_2_HPO_4_ (Sigma-Aldrich, St. Louis, MO, USA), and ultrapure water (Milli-Q system, Millipore, Bedford, MA, USA). Hanks’ Balanced Salt Solution (HBSS 1×, without Calcium, Magnesium, and Phenol Red; Corning^®^, Somerville, MA, USA) was also used during cell culture.

### 2.2. In Vitro Contact Lens Characterization

The characterization of the contact lenses was conducted using eight different contact lens materials (Table 1), including conventional hydrogels and silicone hydrogels, evaluated at three lens powers (+6.00, −0.50, and −6.00 D). For each material and power, three lenses were analyzed, resulting in a total of 72 lenses. The Physicochemical properties of the SCLs were evaluated before dehydration (control) and after rehydration with the insulin loading solution (1750 μg/mL). A concentration of 1750 μg/mL of insulin was selected, as it corresponds to the upper range of concentration reported in the literature for topical administration [25].

The assessed parameters included water content, physical parameters (diameter and base curve), wettability, visible light transmittance, refractive index, central thickness, and power profile.

#### 2.2.1. Dehydration Process

The SCLs were carefully removed from the blister packs and equilibrated for 24 h in a 24-well plate (Merck, Darmstadt, Germany) containing 1 mL of PBS. After removal from the wells, excess surface PBS was gently blotted using pre-wetted Whatman Grade 1 filter paper to ensure reproducible handling following a previous methodology [26]. Lenses were placed on plastic holders with the anterior surface exposed to air at 37 °C in a laboratory oven (Model 100–800, Memmert, Germany) under 30–40% relative humidity (RH). Lens weights were recorded at baseline (0 h), every 5 min during the first hour, and subsequently at 1.5, 2, 4, 6, 8, 10, and 24 h using a digital analytical balance (Fisher Scientific, Waltham, MA, USA) with a precision of ±0.1 mg. After 24 h of dehydration, the lenses were transferred to fresh wells containing 1 mL of 1750 μg/mL insulin loading solution and incubated for 24 h to allow rehydration.

All contact lenses were labeled, and the investigator performing the measurements was blinded to which lens was being measured. Parameters derived from the dehydration process were expressed as dehydration rate (DR) and valid dehydration (VD) [27].

DR represents the dehydration per time interval for each lens at a given time point. In Equation (1), *WT*_(*n*)_ is the sample weight at time *n*, and *WT*_(*n*−1)_ is the sample weight at time *n* − 1, resulting in negative values.

DR*_n_* (%) = [(WT_(*n*)_ − WT_(*n*−1)_) × 100]/WT_(*n*)_ (1)

VD represents the weight loss of each lens at a given time during the dehydration process, relative to its total weight loss. It is computed using Equation (2), where *WT*_(0)_ is the initial sample weight, *WT*_(*n*)_ is the sample weight at time *n*, and *WT*_(*f*)_ is the final lens weight. Positive values are obtained because this value is calculated with respect to the final weight of the sample.

VD*_n_* (%) = [(WT_(0)_ − WT_(*n*)_) × 100]/(WT_(0)_ − WT_(f)_) (2)

The stabilization time was also assessed and defined as the time point at which the difference between two consecutive VD values was ≤2%.

#### 2.2.2. Water Content

The ability of conventional hydrogel or silicone hydrogel contact lenses to retain water in their equilibrium state is called the equilibrium water content (EWC) [23]. Swelling studies of the soft contact lenses were performed under static conditions over 24 h to determine EWC. Dry lenses (*W_D_*) were initially weighed and subsequently immersed in 1 mL of PBS (as a control) or insulin loading solution (1750 μg/mL) at room temperature. After 24 h, each lens was carefully removed, gently blotted with filter paper to remove surface moisture, and immediately weighed (*W_W_*).

EWC (%) was calculated using Equation (3):

(3) $$EWC(\%)=\frac{W_{W}-W_{D}}{W_{W}}\times 100$$

#### 2.2.3. Physical Parameters

Lens diameter and base curve radius were measured before and after insulin loading using an Optimec Chiltern instrument (Optimec Ltd., Malvern, UK). The lens diameter was measured by projecting the lens image onto a graticule magnified 16×, with a measurement accuracy of ±0.025 mm. The base curve radius was calculated using a cylinder–probe system measuring sagittal height across a 10 mm chord (resolution ±0.02 mm). Measurements were performed in a 0.9% saline solution wet cell, and a tilted graticule with a V-shaped support was used for alignment.

#### 2.2.4. Wettability

Lens wettability was assessed using a non-modified commercial videokeratoscope (Medmont E300, Medmont, Australia) as described previously by Carpena-Torres et al. [28]. SCL wettability was measured before (control) and after insulin loading. Excess surface solution was removed using pre-wetted Whatman^®^ Grade 1 filter paper for 2 s before measurement, and all manipulations were performed with plastic tweezers.

The device projects Placido rings onto the anterior surface of the contact lenses, which are placed on the calibration ball with an 8-mm base curve integrated into the system. The distortion of the Placido rings was analyzed using the Medmont software (version 6.2.6) to calculate the tear film surface quality (TFSQ) index. The TFSQ index indicates the stability and distribution of the liquid film on the contact lens surface: lower values indicate higher wettability of the anterior contact lens surface. Immediately after placing the contact lens centered on the calibration ball, a dynamic topography was performed for 120 s (2 frames per second), obtaining 240 measurements. The parameter analyzed was the mean TFSQ value from the 240 frames for a lens diameter of 7 mm.

#### 2.2.5. Transmittance

Light transmittance (%) was measured using a PowerWave XS2 Microplate Spectrophotometer (BioTek Instruments Inc., Winooski, VT, USA) in a 24-well plate, scanning from 400 to 800 nm at 5 nm intervals [29]. Measurements were performed in triplicate after lens swelling in PBS or insulin solution.

#### 2.2.6. Refractive Index

The refractive index (RI) was measured using an Abbe Refractometer (model NAR-1T Solid; Atago Co., Ltd., Tokyo, Japan) before (hydrated in PBS) and after insulin loading at 20–22 °C. Before each measurement, the lenses were blotted with pre-wetted filter paper. Measurements were performed at 589 nm (sodium D-line), with an accuracy of ±0.0002. The hydrated individual lenses were placed directly on top of the holder, which was attached to an appropriate spring to create a force to push the lens into contact with the prism.

#### 2.2.7. Central Thickness

The central thickness was measured in vitro using a Spectral Domain Optical Coherence Tomography (OCT iVue-100, Optovue Inc., Fremont, CA, USA), with the system modified by a custom-designed support that mimics the human ocular surface following the methodology of previous studies [30,31]. The central thickness was measured before (control) and after loading the SCLs with the insulin solution. Each contact lens was placed in a calibrated 8 mm diameter spherical support marked with three white reference points for proper positioning and a circular outline corresponding to the lens diameter to ensure correct alignment. For each contact lens, insulin three independent measurements were taken using the OCT system’s caliper tool. Measurements were corrected for the refractive index of the lens material, since the OCT software (version 2018.1.1.60) assumes a corneal refractive index of 1.376. Central thickness was calculated from the optical path length (OPL) using Equation (4):

(4) OPL=n·L

where *n* represents the refractive index of the medium, and *L* denotes the physical path length of the light within that medium.

#### 2.2.8. Power Profile

Power profiles were measured using a NIMO TR1504 (Lambda X-Ophthalmics, Nivelles, Belgium) instrument before (control) and after loading the SCLs with the insulin solution, following the methodology previously described by Bodas-Romero et al. [32]. The NIMO TR1504 operates based on quantitative deflectometry and Schlieren phase shift analysis [33,34]. This technique detects light ray deviations to accurately determine the optical power of rigid lenses or SCLs immersed in saline solution (λ = 546 nm).

Before each measurement, calibration was performed and lens parameters (refractive index, base curve radius, diameter, and central thickness) were entered. Lenses were placed in a quartz wet cell filled with 0.9% saline solution (*n* = 1.334), with the posterior surface facing downward and centered using the live image provided by the software.

### 2.3. Insulin Loading and Release—In Vitro Studies

The SCL materials that best maintained their physicochemical properties following loading with an insulin solution (1.75 mg/mL, diluted in PEG 400-propylene glycol eye drops) were Nesofilcon A, Stenfilcon A, and Delefilcon A (Table S1 in Supplementary Materials). These materials showed the least pronounced changes, particularly in power profile and wettability, while maintaining all properties within the tolerance limits established by the ISO standard. Therefore, they were selected for subsequent drug release experiments [35]. A total of 27 lenses with a nominal power of −0.50 D were used for each concentration.

Prior to drug loading, the SCLs were removed from their original blisters and placed in individual wells of a 24-well plate containing 1 mL of phosphate-buffered saline (PBS, pH 7.4). The lenses were incubated for 24 h at room temperature to equilibrate and remove any residual storage solution, which could differ among manufacturers. Subsequently, the lenses were removed, placed in plastic holders, and dehydrated in an oven at 37 °C for 24 h. For drug loading, each dehydrated lens was placed in a well containing 1 mL of insulin loading solution (875 or 1750 µg/mL) and incubated for 24 h. At predetermined time points (1, 4, 6, and 24 h), aliquots of the loading solution were collected to determine the remaining insulin concentration. Corrections were made to account for sampling during the experiment. The amount of insulin loading into the hydrogels was estimated from the difference between the initial and remaining amounts of insulin at each time point and normalized to the dry weight of each hydrogel. Following the loading phase, the SCLs were gently blotted with filter paper and transferred to new wells containing 1 mL of 0.9% saline solution at 34.5 °C under gentle shaking to evaluate drug release. Sink conditions were maintained throughout the release experiments by periodically replacing the sampled medium with fresh release medium. Specifically, 100 µL aliquots were withdrawn at each sampling point during the first 8 h and immediately replaced with an equal volume of fresh medium.

The concentrations of insulin in both the loading and release media were determined by UV–visible spectrophotometry at a wavelength of 276 nm using a 96-well quartz microplate and a PowerWave XS2 Microplate Spectrophotometer (BioTek Instruments, Winooski, VT, USA). This wavelength was selected based on a prior spectral scan of insulin performed between 250 and 400 nm, which identified a maximum absorbance peak at 276 nm (Figure S1 in Supplementary Materials). Quantification was carried out using a calibration curve (1–50 µM) obtained from different dilutions of an insulin stock solution (Figure S2 in Supplementary Materials). Finally, to estimate the affinity of insulin for the polymer network, the network/water partition coefficient (K_N/W_) was determined for each hydrogel. K_N/W_ was calculated as the difference between the total amount loaded and the amount that could be hosted in the aqueous phase (estimated from water uptake values) and divided by the concentration of insulin in the loading solution [36].

Water uptake (%) was calculated using Equation (5):

(5) $$Water\;uptake\left(\%\right)=\frac{W_{W}-W_{D}}{W_{D}}\times 100$$

where *W_D_* and *W_W_* represent the weights of the dry and hydrated lenses, respectively.

### 2.4. Drug Content

To assess the drug content in the SCLs with a 10 mm diameter, six samples per material were analyzed. The lenses were cut to a diameter of 10 mm using previously sterilized biopsy punches. The SCLs were loaded with insulin solutions at 875 and 1750 µg/mL concentration for 24 h, as previously described in Section 2.3. Subsequently, they were immersed in 1 mL of HBSS for 24 h at 34.5 °C under gentle shaking. After 24 h, insulin content was measured by spectrophotometry microplate reader Multiskan SkyHigh (Thermo Fisher Scientific, Wilmington, DE, USA).

### 2.5. Cell Culture

Human corneal epithelial cells (HCE-2, 50.B1, CRL-11135, American Type Culture Collection, Manassas, VA, USA) were grown at 37 °C in humidified air with 5% CO_2_, in a suitable standard culture medium. The standard medium comprised a keratinocyte serum-free medium kit, 5 ng/mL human recombinant epidermal growth factor, and 0.05 mg/mL bovine pituitary extract (Gibco^TM^, Thermo Scientific^TM^, Waltham, USA), which was supplemented with 5 mL of 500 ng/mL hydrocortisone (BioReagent, Sigma-Aldrich), 2.5 mL of 100 IU penicillin-streptomycin (BioReagent, Sigma-Aldrich), 10 µg gentamicin (BioReagent, Sigma-Aldrich), 1 mL of amphotericin B (BioReagent, Sigma Aldrich), and 250 µL of 5 ng/mL insulin (from bovine pancreas, BioReagent, Sigma-Aldrich). The culture medium was replaced three times per week, and the cells were cultured on surfaces previously coated with 100 µL/cm^2^ collagen solution (type I from rat tail, BioReagent, Sigma-Aldrich) [37]. Cells were cultured submerged for 7–10 days and thereafter cultured at the air-liquid interface.

#### 2.5.1. Corneal Permeability Assay

Permeability studies were assessed using HCE-2 cell suspensions at a density of 100,000 cells/cm^2^. The cells were seeded onto a previously collagen-coated polyester membrane (0.4 μm pore size, 1.12 cm^2^ growth area, Transwell^®^, Corning^®^, Somerville, USA) and grown in an incubator for 28 days following the protocol described in Section 2.5, with 1.5 mL of the standard medium in the basolateral chamber and 0.5 mL in the apical chamber, replaced twice per week. Throughout the 28-day culture period, the cultures were visually inspected at each scheduled medium change for signs of contamination, including turbidity, color change, and abnormal odor. The study was designed to determine the permeation of insulin from the apical chamber, acting as the donor, to the basolateral chamber, acting as the receiver, through the corneal epithelial cell layer.

Transepithelial electrical resistance (TEER) is an important indicator of epithelial barrier integrity [38,39]. TEER was measured before and after the permeability assay using an epithelial voltohmmeter (EVOM3, World Precision Instruments, Sarasota, FL, USA) equipped with STX2 electrodes. Values were corrected for blank resistance and normalized to the surface area (Ω·cm^2^).

The SCLs were cut with autoclaved biopsy punches (Ø = 10 mm) and placed in the donor chamber containing the cells. To avoid potential interference caused by direct physical contact between the cells and SCLs under evaluation, a modified permeability setup was developed in which a spatial separation was introduced between them. A silicon ring (11 mm internal diameter, 2 mm width) was sterilized by immersion in absolute ethanol followed by overnight UV light exposure, then carefully placed on top of the cell layer. The apical chamber was prefilled with 0.5 mL of standard medium without insulin supplementation to ensure complete immersion of the experiment setup, while 1.5 mL was placed in the basolateral chamber. Insulin was not included in the standard medium to prevent interference with the quantification of insulin permeating across the epithelial barrier.

The SCLs were carefully placed on top of the silicon ring in the donor chamber, ensuring complete coverage of the medium at the start of the experiment. At each time point (0.5, 1, 2, 3, 4, 5, 6, 7, 8, and 24 h), 200 μL of medium was withdrawn from the receptor chamber and replaced with an equal volume of fresh standard medium. The experiment was conducted under optimal cell growth conditions (27–28 days) to minimize any cell loss and consequent alterations in membrane permeability. To determine the amount of insulin permeated, samples from the basolateral chamber were collected and analyzed by UV-Vis spectrophotometry at 276 nm using a 96-well quartz microplate and a microplate reader Multiskan SkyHigh (Thermo Scientific, USA), as previously described.

In addition, the apparent permeability coefficient (Papp) was determined. Papp is defined as the rate of drug permeation across a biological membrane normalized to the membrane surface area (1.12 cm^2^) and the initial drug concentration [40]. It was calculated according to the following equation:

(6) $$Papp=\;\frac{J}{CD\;\times \;S}$$

where *J* represents the transmembrane flux of insulin from the donor to the receptor chamber, obtained as the slope of the cumulative permeated amount versus time plot. *CD* is the initial drug concentration in the donor chamber, and *S* is the effective surface area of the membrane.

#### 2.5.2. Cell Viability Assay

Corneal epithelial cytotoxicity was performed in HCE-2 cells using the MTT (Thiazolyl Blue Tetrazolium, Bromide, BioReagent, Sigma-Aldrich) assay. Cells were resuspended, seeded into 24-well plates (100,000 cells/cm^2^ density), and incubated under the growing conditions previously described in Section 2.5. After overnight growth and 80–90% confluence, the cells were exposed to SCLs loaded with insulin solutions (875 and 1750 µg/mL), unloaded SCLs, or standard medium (control) for 24 h. The duration of the experiment was based on the duration of permeability experiments and the expected residence time of the SCLs on the eye. Additionally, a serial dilution of insulin in standard medium was prepared to determine the effect of the free drug on cell viability, with concentrations ranging from 3500 µg/mL to 13.67 µg/mL.

To prevent physical contact between the cells and SCLs, a silicon ring was carefully placed on the cell layer after adequate sterilization, and the SCL was carefully positioned on top of the ring (Section 2.5.1). After 24 h of incubation, the plates were carefully emptied, and the cell layers were washed with HBSS. Cells were further incubated with MTT in standard medium (to a final concentration of 1 mg/mL) for 2 h. Upon removal of the MTT solution, cells were lysed by exposure to dimethyl sulfoxide (DMSO, Thermo Scientific™, Waltham, USA) for 30 min at 37 °C under shaking. The absorbance of formazan was measured at 590 nm using a Multiskan SkyHigh microplate reader (Thermo Scientific, USA). Cell viability after exposure to insulin solutions or drug-loaded SCLs was expressed as a percentage relative to control cells cultured in the standard medium.

### 2.6. Statistical Analysis

Statistical analysis was performed using IBM SPSS Statistics (V 26, IBM, New York, NY, USA). Data are expressed as mean ± standard deviation (SD). Normality was assessed using the Kolmogorov–Smirnov test. For normally distributed data, comparisons between two related samples were carried out using the paired Student’s *t*-test. For non-normally distributed data, nonparametric tests were applied, including the Wilcoxon signed-rank test for related samples and the Mann–Whitney U test for independent groups. For multiple group comparisons, the Friedman test was used for related samples and the Kruskal–Wallis test for independent samples. A *p*-value < 0.05 was considered statistically significant.

## 3. Results and Discussion

### 3.1. Dehydration Process and Water Content

Water content is an important parameter related to the comfort of SCLs and is closely associated with their dehydration behavior [41]. The degree of dehydration and the time required to reach equilibrium are influenced by several factors, including temperature and relative humidity [42], as well as intrinsic lens properties such as the maximum water content of the polymer [43] and lens thickness [44,45]. In the present study, dehydration and EWC were determined using the standard gravimetric oven-drying method, which has been widely employed in previous studies [46,47,48].

All contact lenses were grouped according to the back-vertex power (−6.00, −0.50, and +6.00 D) and material for the dehydration analysis. All measurements were conducted under controlled environmental conditions inside the oven, with constant relative humidity (38.01 ± 0.02%) and temperature (37.11 ± 1.28 °C). Under these standardized conditions, any differences observed in dehydration behavior can be attributed exclusively to the contact lens material.

**Figure 1:**
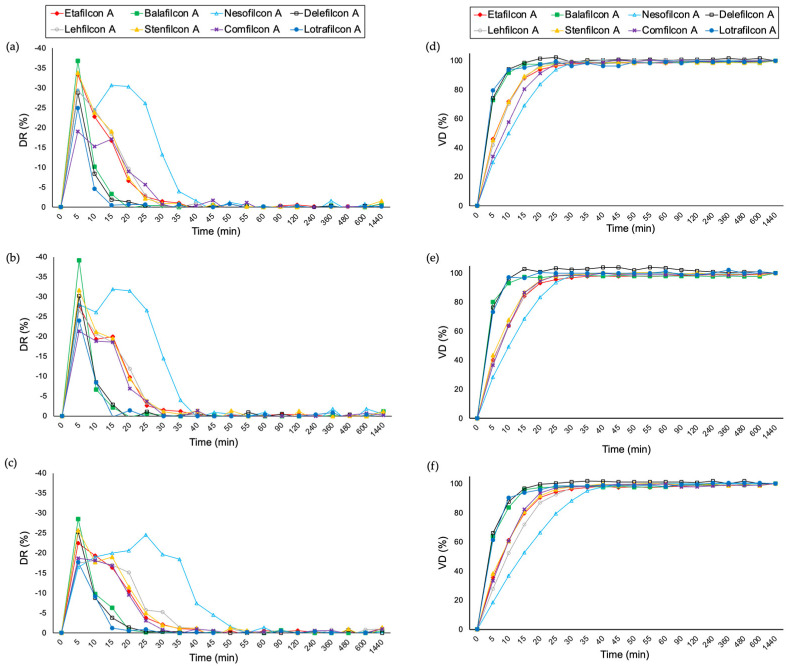
Dehydration ratio (DR) for contact lenses with dioptric powers of +6.00 D (**a**), −0.50 D (**b**), and −6.00 D (**c**), and valid dehydration (VD) for lenses with dioptric powers of +6.00 D (**d**), −0.50 D (**e**), and −6.00 D (**f**), for each material during the dehydration process.

Figure 1 shows DR and VD results throughout the 24-h dehydration process. All tested materials reached stabilization within the first hour of this process (Table 2). During the initial 5 min, Balafilcon A and Stenfilcon A exhibited the highest DR values across all lens powers. For +6.00 D lenses, DR values were −36.80 ± 11.45% for Balafilcon A and −33.82 ± 10.40% for Stenfilcon A. For −0.50 D lenses, DR values reached −39.16 ± 7.38% and −31.65 ± 11.25%, respectively, for the same materials. The lowest DR values for these materials were observed in −6.00 D lenses (−28.54 ± 3.65% for Balafilcon A and −25.71 ± 1.67% for Stenfilcon A). In contrast, Nesofilcon A consistently exhibited the lowest DR and VD values and showed the longest time to reach stabilization. In particular, Nesofilcon A lenses with −6.00 D power presented the lowest VD during the first 5 min of dehydration (18.65 ± 3.15%). Conversely, Balafilcon A lenses with −0.50 D power showed the highest VD value (80.11 ± 7.87%) among all tested materials and powers.

Jones et al. [49] demonstrated a strong positive correlation between dehydration time and EWC, showing that longer dehydration times are associated with higher EWC values. This trend is consistent with the present findings, where Nesofilcon A (78%) showed a longer stabilization time compared with Balafilcon A (36%) and Lotrafilcon A (24%). While some studies have reported lower dehydration rates in silicone hydrogel materials due to the hydrophobic nature of the silicone matrix [27,50,51], others, such as González-Méijome et al. [27], observed no significant differences between silicone hydrogel and conventional hydrogel lenses when similar EWCs are considered. Overall, these findings suggest that while the polymer structure determines the ratio of bound-to-free water, the bulk EWC appears to be the primary factor governing dehydration behavior and stabilization time, in agreement with the present study. Furthermore, the dehydration process observed in this study was more pronounced than that reported in a previous study [52], likely due to the experimental conditions employed, especially the elevated temperature of 37 °C.

Finally, a limitation of this dehydration process was the use of a temperature of 37 °C and a relative humidity of 30–40%, as these conditions do not fully reflect the physiological ocular environment. Different dehydration conditions may influence the absolute dehydration rates and stabilization times reported. However, all materials were evaluated under identical experimental conditions, allowing direct comparison among them.

The EWC results of lenses immersed in PBS (control) and lenses loaded with the insulin solution are summarized in Table 3. The measured values are in good agreement with the manufacturers’ specifications (Table 1). Upon comparison of EWC values before and after insulin loading, statistically significant differences were observed for the CH materials Etafilcon A (*p* = 0.001) and Nesofilcon A (*p* = 0.04). Similarly, significant differences were observed for the SH materials Balafilcon A (*p* = 0.001) and Delefilcon A (*p* = 0.009), which possess comparable core water contents of 33% in the case of Delefilcon A and 36% for Balafilcon A.

The difference in EWC before and after loading the lenses with the insulin solution is a critical parameter for maintaining the physicochemical properties of SCLs within the tolerance limits established by ISO 18369-2:2017 [35], which specifies an acceptable EWC variation of ±2% relative to the control value (PBS). In this study, the tolerance was exceeded only for Etafilcon A and Balafilcon A, which exhibited an approximate difference of 3%. For the remaining materials studied, the EWC variation remained within the ±2% limit, in agreement with previously reported results for drug-loaded contact lenses [53,54]. For example, Desai et al. [53] investigated the release of timolol and hyaluronic acid (HA) from semi-circular ring-implanted contact lenses for glaucoma treatment and observed a slight reduction in EWC in lenses soaked with timolol compared to HA. Likewise, in studies addressing the treatment of bacterial conjunctivitis, minimal differences in EWC, remaining within ISO tolerance limits, were reported for SCLs loaded with hydrophilic drugs such as moxifloxacin hydrochloride and HA [54], showing behavior comparable to that observed for insulin-loaded SCLs, attributable to the hydrophilic nature of these drugs.

### 3.2. Physical Parameters

The results for physical parameters, including base curve radius and diameter, are presented in Table 4. No statistically significant differences were observed in either the base curve radius (*p* ≥ 0.08) or the diameter (*p* ≥ 0.32) before and after loading the SCLs with the insulin solution, regardless of the material tested, indicating that the treatment did not compromise their dimensional stability. These findings were further confirmed by comparison with ISO 18369-2:2017 [35], which establishes permissible variation limits of ±0.20 mm for both base curve radius and total diameter relative to the control conditions (PBS). All evaluated materials complied with the ISO-specified tolerance limits. Additionally, for appropriate clinical performance, SCLs should ensure complete corneal coverage in all gaze positions and during blinking. Accordingly, the typical diameter range for SCLs is 13.8–14.5 mm [55]. All tested lenses, both before and after insulin loading, remained within this range. Although the present study was conducted in vitro, these dimensional parameters remain relevant in view of potential future clinical applications, as they are directly associated with safety and wearer comfort [56]. Furthermore, an appropriate base curve radius is essential to maintain adequate lens movement and fitting characteristics on the ocular surface; alterations in this parameter may compromise lens adaptation and overall fitting performance [57].

### 3.3. Wettability

Wettability is a key surface-related property of SCL materials, as it reflects the ability of the lens surface to sustain a stable aqueous film [58,59]. The Medmont E300 videokeratoscope enables the indirect assessment of SCL surface wettability through optical analysis of the regularity of the pre-lens tear film pattern. At present, no ISO standard is available for the in vitro assessment of wettability in SCLs. Nevertheless, videokeratoscopy has been widely used in earlier studies to evaluate SCL wettability [28,60].

Wettability results before and after loading the SCLs with the insulin solution are presented in Table 3. Statistically significant differences were observed for Etafilcon A (*p* = 0.002) and Lotrafilcon A (*p* = 0.007) when comparing lenses before (control) and after insulin loading. For Etafilcon A, insulin loading resulted in a significant improvement in wettability, as evidenced by a decrease in the TFSQ mean value from 0.710 ± 0.266 (control) to 0.325 ± 0.135 (insulin). This improvement may be attributed to its inherently hydrophilic polymer matrix, which facilitates the retention of hydrophilic excipients (PEG 400 and propylene glycol) from the loading solution, thereby enhancing surface hydration and tear film stability [61]. In contrast, Lotrafilcon A showed a statistically significant worsening of wettability, which may be attributed primarily to prior immersion in PBS for 24 h, leading to a modification of the plasma-treated, SmartShield-modified surface and a consequent reduction in baseline hydrophilicity. This interpretation is compatible with the reported difficulty in maintaining a stable, highly hydrophilic plasma-modified surface on silicone hydrogel materials, whose polar groups are highly sensitive to environmental changes [62]. Although no statistically significant changes were detected in the remaining materials (Balafilcon A, Delefilcon A, Stenfilcon A, and Comfilcon A), these lenses showed a tendency toward improved wettability after insulin loading. This trend suggests that the incorporation of the insulin solution, formulated in PEG 400–propylene glycol eye drops, may enhance surface hydration.

Overall, the results are in line with previous studies indicating that the presence of hydrophilic constituents can modulate the wettability of SCL materials. For example, Marx and Sickenberger [60] reported increased wettability for four SCL materials after pre-soaking in a multipurpose disinfecting solution containing two wetting agents (HydraGlyde^®^ and polyethylene glycol), compared with saline solution (control). Carpena-Torres et al. [28] also demonstrated that hyaluronic acid at different concentrations significantly enhanced the in vitro wettability of Oculfilcon D and Somofilcon A compared with saline solution.

### 3.4. Transmittance

Optical transparency is one of the most essential properties of SCLs, as spectral transmittance directly influences visual performance [63]. This property is particularly relevant in drug-loaded SCLs since modifications to the CH or SH SCL matrix arising from drug incorporation or increased drug concentration may adversely affect light transmission within the visible spectrum [64,65]. Spectral transmittance is generally expressed as the percentage of transmitted light across the visible electromagnetic spectrum. As extensively reviewed by Efron and Maldonado-Codina [66], hydrogel materials intended for use as SCLs should transmit more than 90% of visible light to ensure adequate optical performance.

**Figure 2:**
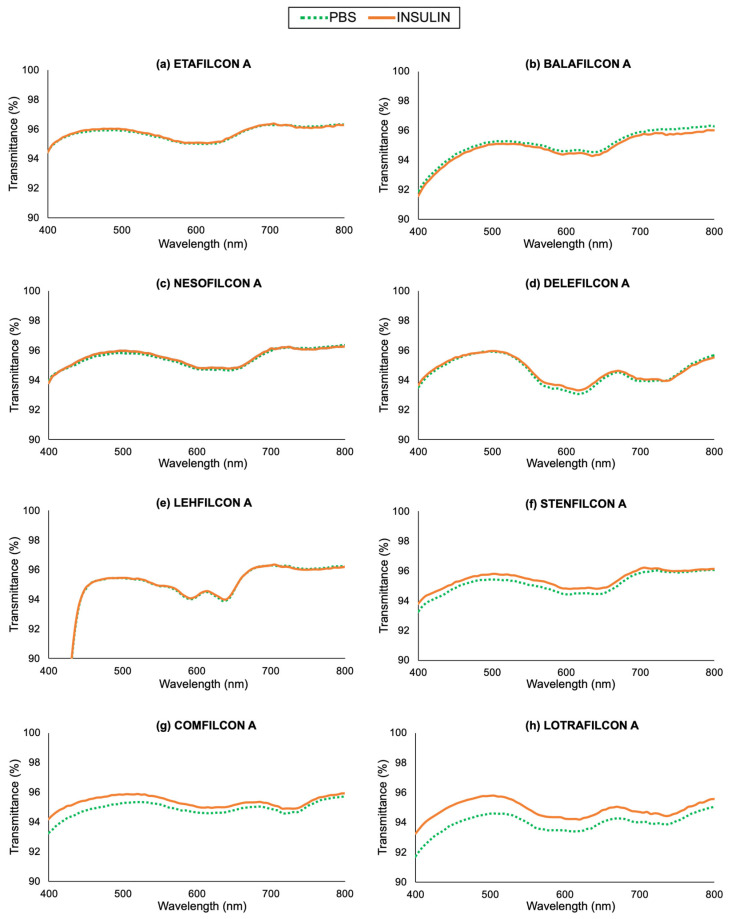
Light transmittance (%) of the different contact lens materials (**a**–**h**) in the 400–800 nm spectral region. The green dashed line represents contact lenses immersed in the control solution (PBS), whereas the orange solid line represents insulin-loaded contact lenses.

The spectral transmittance results are shown in Figure 2. All evaluated materials exhibited light transmission above 90% in the wavelength range from 435 nm to 800 nm. However, according to manufacturer specifications, Lehfilcon A is designed to reduce high-energy visible light (HEVL) reaching the posterior segment of the eye by approximately 34% in the 380–450 nm range [67]. Consequently, a reduction in transmittance below 90% was observed between 400 and 430 nm for this material in both control and insulin-loaded lenses.

According to the ISO standard [35], the acceptable tolerance for spectral transmittance in the visible region is ±5% in absolute terms. This criterion was satisfied by all materials evaluated in the present study, except for Lehfilcon A. Notably, this deviation is intrinsic to the material’s optical design and independent of insulin loading.

### 3.5. Central Thickness and Refractive Index

The results of central thickness and refractive index (RI) are presented in Table 5. Central thickness data were stratified by both material and lens power, as this parameter varies depending on whether the lens power is positive or negative [55]. In contrast, RI results are reported as a function of lens material, as this parameter is an intrinsic property of the polymer composition and is not expected to vary with lens power.

Variations in central thickness may influence lens functionality, making its evaluation essential for optimizing SCL performance. OCT is a non-invasive, high-resolution imaging technique widely used in ophthalmology to assess the thickness of ocular structures [68]. In recent years, its application in material science has expanded, particularly for evaluating contact lens fitting and thickness [69]. In the present study, central thickness was measured using an eyeball support model designed to closely mimic the ocular surface, following the methodology of previous studies [30,31].

Statistically significant differences in central thickness were observed only for Etafilcon A (*p* = 0.042), which exhibited a slight increase across all lens powers following insulin loading. ISO 18369-2:2017 [35] specifies acceptable tolerance limits of ±10 µm for thin SCLs (≤100 µm) and ±15 µm for thicker SCLs (>100 µm). All materials evaluated in this study showed thickness variations below these thresholds (<10 µm or <15 µm, depending on the initial thickness), indicating compliance with ISO standards. Previous studies evaluating central thickness before and after drug loading have similarly reported minimal changes within ISO-accepted limits [31,70], further supporting the findings of the present work. However, it is worth noting that most previous studies investigating drug delivery using SCLs have performed physical, chemical, or mechanical characterization but did not include central thickness measurements [71,72,73,74,75].

Refractive index is defined by ISO 18369-1:2017 [24], as the “ratio of the speed of light in a vacuum to the speed of that same light in a material”. Although RI is considered an essential property to be evaluated in commercial SCLs according to the ISO standard [23], to the best of the authors’ knowledge, it has been scarcely investigated in therapeutic SCLs. Faria et al. [31] evaluated refractive index changes in SCLs before and after loading with a resveratrol solution and reported no clinically relevant variations in SH lenses, although significant changes were observed in CH SCLs. Nevertheless, that study assessed only one CH and SH material. In contrast, the present study evaluated two CH materials and six SH materials, providing a broader comparative analysis. Statistically significant differences were observed for Nesofilcon A, Delefilcon A, Stenfilcon A, and Comfilcon A (*p* ≤ 0.016) after insulin loading, with the largest variation observed for Comfilcon A (SH), reaching a maximum change of 0.005. However, despite statistical significance, these changes were not clinically relevant, as all values remained within the ISO tolerance limit for RI variation (±0.005) [35].

### 3.6. Power Profile

**Figure 3:**
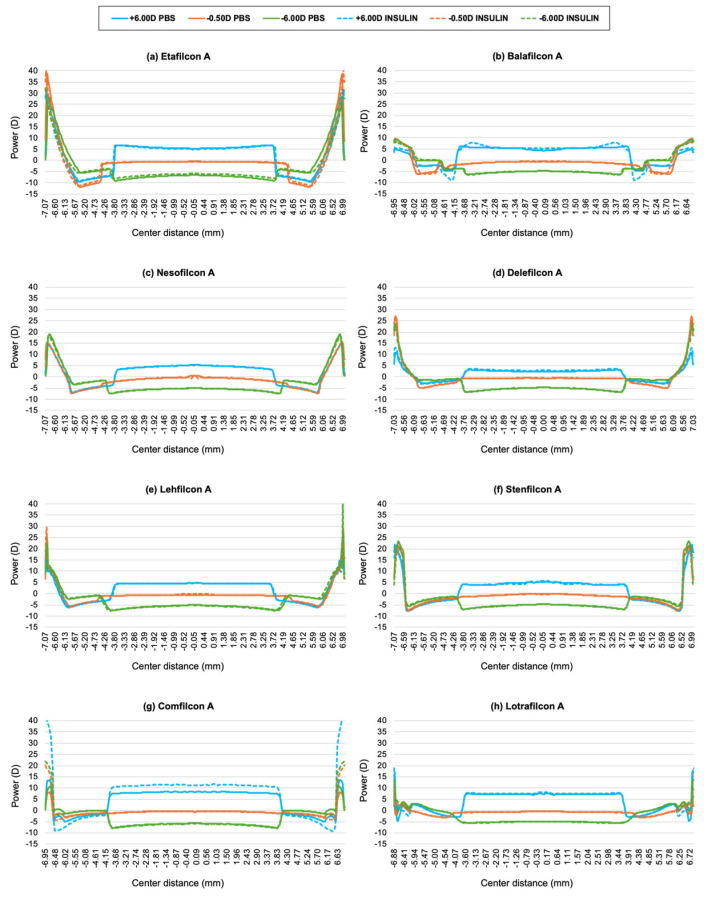
Power profiles (D) of the different contact lens materials (**a**–**h**) at optical powers of −6.00, −0.50, and +6.00 D, soaked in PBS (control) and loaded with insulin.

The power profiles of each material and nominal power before and after loading with insulin are shown in Figure 3. To enable a more direct comparison, the differences in refractive power between insulin-loaded and PBS-soaked (control) lenses at specific distances from the center of the SCL are presented in Table 6. Four measurement points were located within the optical zone (OZ) of the lens (−3.0, −0.5, +0.5, and +3.0 mm), and two additional points were positioned approximately in the peripheral zone (PZ) (−6.0 and +6.0 mm).

ISO 18369-2:2017 defines optical tolerances for SCLs with a back-vertex power ≤ 10.00 D as ±0.25 D [35]. This threshold is consistent with clinical perception, as differences ≤ ±0.20 D are generally imperceptible to patients, whereas refractive changes ≥ ±0.25 D may be considered clinically significant [76]. The mean differences at −3.0 and +3.0 mm from the lens center for the +6.00 D power exceeded the ±0.25 D tolerance limit for Balafilcon A, Delefilcon A, Comfilcon A, and Lotrafilcon A. In contrast, for the −6.00 D power, only Etafilcon A exceeded the ISO tolerance limit (0.96 D) at these positions. At −0.5 and +0.5 mm distances from the lens center, Nesofilcon A and Stenfilcon A complied with the ISO tolerance limits across all tested powers. Balafilcon A, Delefilcon A, Lehfilcon A, and Lotrafilcon A also complied with the ISO tolerance limits for the −6.00 D power within the same positions, while Lehfilcon A additionally complied for the +6.00 D power. For lenses with a nominal power of −0.50 D, all materials complied with the ISO tolerance limits within the OZ, except for Lehfilcon A. At peripheral positions (±6.0 mm), Etafilcon A and Balafilcon A exhibited the greatest refractive power differences across all lens powers.

In optometry, power profile analysis is widely used in the design and characterization of multifocal SCLs for myopia control [32,77,78]. However, to the best of the authors’ knowledge, this is only the second study evaluating power profiles of different SCL materials and nominal powers before and after drug incorporation into the lens matrix. In a previous study, Faria et al. [31] reported that after loading −0.50 D SCLs with a resveratrol solution, no differences greater than ±0.25 D were observed within the central 6 mm (OZ), except for Somofilcon A and Senofilcon A materials.

### 3.7. Insulin Loading and Release—In Vitro Studies

Following the dehydration-based physicochemical characterization of the SCLs, three materials were selected for insulin loading and release studies: Nesofilcon A, Stenfilcon A, and Delefilcon A. These materials were selected based on their superior performance during physicochemical characterization. All variations observed following insulin loading, relative to the control, remained within the tolerance limits established by the ISO 18369-2:2017 for SCLs [35]. Although a previous study evaluating melatonin-loaded SCLs reported no significant effect of dioptric power on drug delivery, all lenses used in these experiments had the same refractive power (−0.50 D) to minimize potential variability associated with lens thickness [79].

Among the available strategies for drug incorporation into SCLs, the soaking method was selected due to its simplicity and cost-effectiveness [80,81]. SCLs were first equilibrated in PBS, subsequently dried, and then immersed in different insulin loading solutions (1750 µg/mL and 875 µg/mL) prepared in PEG 400-propylene glycol (Systane Ultra^®^) eye drops to evaluate drug loading capacity. All SCLs were soaked for at least 24 h. The loading solution was prepared using commercial eye drops containing PEG 400 and propylene glycol (Systane Ultra^®^) to simulate a clinically relevant vehicle for ocular insulin delivery. Since this vehicle was identical for all tested formulations, the differences in loading capacity are strictly attributed to the drug–polymer matrix interactions.

**Figure 4:**
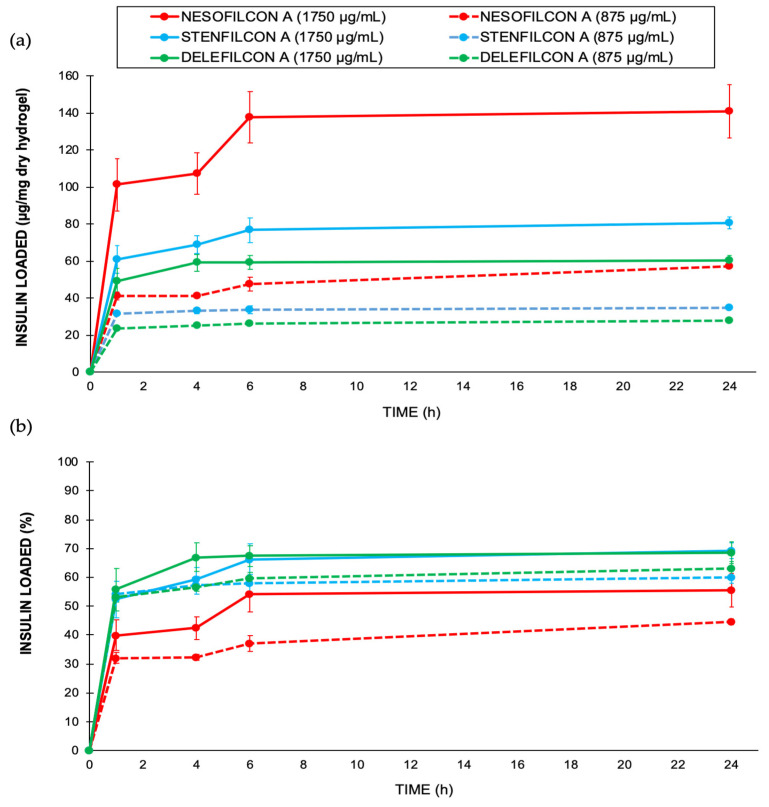
Insulin loading at 1750 µg/mL (solid line) and 875 µg/mL (dashed line) in Nesofilcon A (red), Stenfilcon A (blue), and Delefilcon A (green) SCL materials after 1, 4, 6, and 24 h, shown as µg/mg of dry hydrogel (**a**) and percentage relative to the total amount loaded into each material (**b**).

As expected, the amount of insulin loaded into the hydrogels increased with increasing loading concentration (Figure 4a,b). Equilibrium loading was achieved within 6 h for most materials. However, for Nesofilcon A at the lower insulin concentration (875 µg/mL), statistically significant differences were observed in the amount loaded at different time points (*p* ≤ 0.039), indicating that equilibrium had not yet been fully established after 24 h.

Notably, the amount of insulin loaded per mg of dry hydrogel (Figure 4a) was significantly higher for the CH material Nesofilcon A than for the SH materials Stenfilcon A and Delefilcon A at both insulin concentrations after 24 h (*p* < 0.001). This behavior can be attributed to the higher hydrophilicity of Nesofilcon A, which contains N-vinyl-2-pyrrolidone (NVP) and poly(vinylpyrrolidone) (PVP), as well as to its substantially greater swelling capacity (363.7%) compared with Stenfilcon A (129.9%) and Delefilcon A (48.3%). Consistent with these observations, Nesofilcon A exhibited a network/water partition coefficient (K_N/W_ = 62–77), approximately two-fold higher than that recorded for Stenfilcon A (K_N/W_ = 39–45) and Delefilcon A (K_N/W_ = 32–34), indicating a greater affinity of insulin for the hydrogel network.

Among the SH materials, Stenfilcon A showed a significantly higher insulin uptake than Delefilcon A (*p* < 0.001). Although Delefilcon A incorporates a highly hydrophilic surface treatment [82], its silicone-rich core remains comparatively more hydrophobic than that of Stenfilcon A, resulting in a lower swelling capacity and, consequently, a reduced insulin loading capacity.

In contrast, when loading was expressed as the percentage of insulin retained by the entire lens after 24 h (Figure 4b), SH lenses exhibited higher values than the CH material. This difference is mainly attributable to the substantially greater mass of the SH lenses. Accordingly, SH lenses retained more than 59% of the insulin initially present in the loading solution at both concentrations after 24 h, whereas Nesofilcon A retained 55.59 ± 5.85% and 44.63 ± 0.95% of the initial insulin at loading concentrations of 1750 and 875 µg/mL, respectively, as estimated from the decrease in insulin amount in the loading medium over the loading period.

Insulin-loaded SCLs were immersed in 1 mL of 0.9% saline solution maintained at 34.5 °C, corresponding to the physiological temperature of the ocular surface, for release studies [83]. Although reliable in vitro release models capable of accurately predicting in vivo drug release from SCLs have not yet been fully established [84], previous studies have demonstrated that the performance of soaked SCLs as drug delivery systems is strongly influenced by the physicochemical compatibility between the SCL material and the drug [79,81,85,86].

In the present study, saturation of the release medium was prevented by replacing the withdrawn aliquots at predetermined time points with fresh 0.9% saline solution. This volume replacement was considered when calculating the cumulative concentration of insulin released.

**Figure 5:**
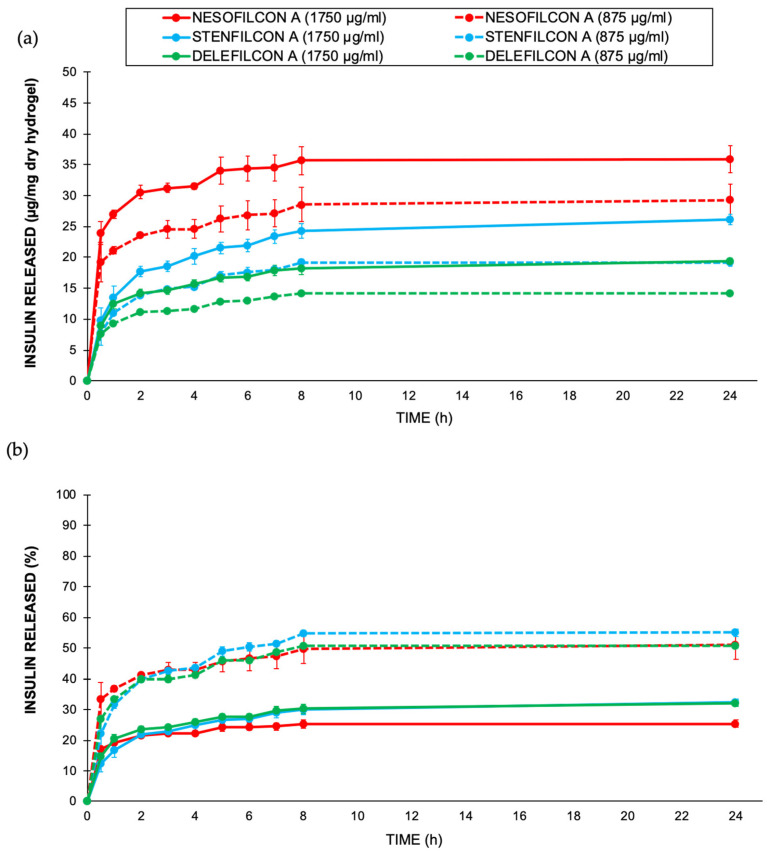
Cumulative insulin released (µg/mg dry hydrogel) (**a**) and percentage of insulin released relative to the total amount loaded into each material (**b**) from Nesofilcon A (red), Stenfilcon A (blue), and Delefilcon A (green) SCLs materials after loading with 1750 µg/mL (solid line) and 875 µg/mL (dashed line) insulin solutions for 24 h.

After 24 h, significantly higher amounts of insulin were released from the CH Nesofilcon A compared with the SH materials at both loading concentrations (*p* < 0.001) (Figure 5a). In contrast, SH lenses, particularly Stenfilcon A, exhibited a more sustained and gradual release profile than Nesofilcon A. This behavior may be attributed to the presence of silicone-based hydrophobic domains within SH matrices (e.g., TRIS-AM and PDMS) [87,88], which may interact with partially exposed hydrophobic regions on the insulin surface, thereby promoting preferential partitioning of the protein within the polymer network and modulating its release kinetics. This interpretation is consistent with a previous report on silicone hydrogel contact lenses, where hydrophobic drugs such as epalrestat have been shown to interact with silicone-rich macromer structures [89].

When release was expressed as a percentage of insulin released from the contact lens (Figure 5b), calculated relative to the amount loaded, no statistically significant differences were observed between CH and SH lenses loaded with 875 µg/mL after 24 h (*p* = 0.301). In addition, when normalized to lens mass, the absolute amount of insulin released per lens (Figure S3 in Supplementary Material) was significantly higher for SH materials compared with Nesofilcon A (*p* < 0.001), reflecting their higher dry mass and consequently a higher total dose per lens.

Overall, lenses loaded with 875 µg/mL showed a higher relative release (approximately 50–55%) than those loaded with 1750 µg/mL (approximately 25–33%). Although higher loading concentrations resulted in greater absolute amounts of insulin released, the relative release efficiency decreased, suggesting a non-linear loading–release relationship. This trend indicates that at higher loading levels, a larger fraction of insulin may remain retained within the hydrogel matrix, likely due to stronger drug–polymer interactions and increased diffusional resistance within the network.

### 3.8. Drug Content

The determination of drug content is an essential procedure in the development and characterization of drug delivery systems [90]. Drug content was quantified in all SCL formulations to evaluate the variability in insulin loading associated with the use of two different initial drug concentrations. The mean drug content values obtained for each material are presented in Table 7. The amount of drug loaded and released into 10 mm diameter SCL discs was determined to subsequently assess cytotoxicity and epithelial permeability. All materials showed a relative standard deviation ≤ 3.52%, indicating good content uniformity. Moreover, no samples exceeded the 5% deviation range from the mean load and release values, demonstrating compliance with accepted uniformity criteria [91].

### 3.9. Cell Viability Assays and Corneal Permeability

The safety of ophthalmic drug delivery systems has been widely evaluated using in vitro models [92]. In particular, immortalized corneal epithelial cell lines, such as HCE-2, are considered a reliable alternative to in vivo studies and are especially valuable during the early stages of formulation development [93]. These models mimic the corneal epithelial barrier and have been employed to evaluate toxicity and penetration of several pharmaceutical compounds [93].

In this study, to prevent direct contact between the SCLs and the epithelial cells, a modified experimental setup was implemented by incorporating a silicone ring to maintain a physical separation between the lenses and the cell layer. This separation is likely greater than that occurring under physiological in vivo conditions, which may result in an increased diffusion distance and consequently a delay in drug diffusion time.

The 28-day culture model carries an inherent risk of contamination and cell variability. However, continuous antibiotic supplementation, the absence of any visual indicators of microbial growth throughout the culture period, and TEER values remaining within the expected physiological range collectively support the maintenance of culture integrity and indicate that transport measurements were not compromised. TEER values were 191.27 ± 9.33 Ω·cm^2^ before the permeability assay and 221.01 ± 8.47 Ω·cm^2^ after 24 h. The TEER values recorded prior to the permeability assay are consistent with the range reported by Becker et al. for validated immortalized human corneal epithelial cell line models, who noted values of ≤100–200 Ω·cm^2^ to be characteristic of such systems [94]. Whilst modestly lower than the 246 ± 7 Ω·cm^2^ reported by Bíró et al. for HCE-T cells under a comparable submerged-to-ALI protocol [95], the values recorded are consistent with a confluent, functionally intact layer appropriate for drug transport screening. The permeation data reported in this study should therefore be interpreted in the context of a validated in vitro screening platform designed to identify lead contact lenses and insulin concentrations for progression to in vivo animal studies, where the full complexity of the native corneal barrier can be assessed.

Furthermore, the physiological relevance of the model is further supported by the post-experiment TEER, which increased significantly following the 24-h permeability assay (*p* = 0.008). This finding is consistent with Bíró et al. [95], who similarly reported a TEER increase during corneal permeation experiments using HCE-T cells, interpreting this as confirmation of the absence of barrier damage. A further hypothesis of insulin increasing tight junction integrity of brain cell lines as reported by Ito et al. [96]. Collectively, the pre- and post-experiment TEER data confirm that the epithelial barrier remained intact throughout, supporting the validity of the insulin transport data and the suitability of lead formulations identified for progression to in vivo investigation.

**Figure 6:**
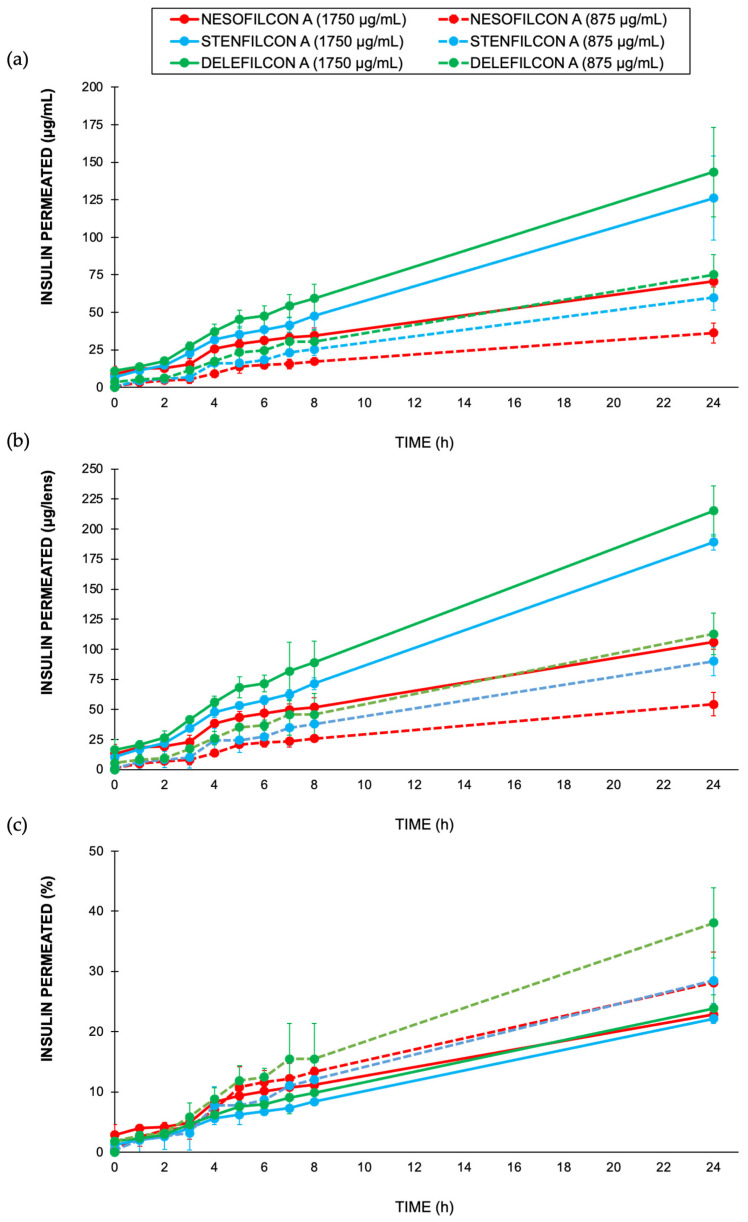
Cumulative insulin concentration in the receptor medium (µg/mL) (**a**), cumulative insulin amount permeated per lens (µg/lens) (**b**), and percentage of insulin permeated (%) (**c**) through the human epithelial corneal cells at different specified times up to 24 h after loading the SCLs with 1750 and 875 µg/mL insulin solution.

The amount of drug loaded into the SCLs influenced the absolute amount of insulin permeated over the testing period (Figure 6a,b), although it did not affect the relative percentage of permeated insulin for Nesofilcon A and Stenfilcon A (Figure 6c). In contrast, Delefilcon A showed a concentration-dependent effect, with a higher percentage of insulin permeation at the lower concentration (38.06 ± 5.85%) than at the higher concentration (23.85 ± 2.31%), indicating higher relative permeation at lower loading levels. Although there is currently no standardized protocol for the topical administration of insulin, previous studies have reported a wide range of therapeutic concentrations, ranging from 0.5 IU/mL to 50 IU/mL [19,25]. The doses permeated from the contact lenses evaluated in the present study fall within this reported range. Nevertheless, further in vivo studies are required to determine the optimal insulin dose for topical ocular delivery and to establish the most effective therapeutic regimen.

Regarding the apparent permeability coefficient, no statistically significant differences were observed among the materials loaded with 1750 μg/mL of insulin, which exhibited values ranging from 1.91 × 10^−6^ to 2.90 × 10^−6^ cm/s (*p* = 0.111). Similarly, no significant differences were found for the SCLs loaded with 875 μg/mL, which showed Papp values between 2.76 × 10^−6^ and 4.28 × 10^−6^ cm/s (*p* = 0.177).

Overall, both loading concentrations enabled insulin permeation across the epithelial cell model, demonstrating its potential to reach corneal exposure levels within this in vitro system. However, given the simplified nature of the HCE-2 model compared with the in vivo ocular environment, these results should be interpreted with caution when extrapolating to clinical conditions. In particular, the model does not reproduce important physiological factors such as tear turnover, blinking, nasolacrimal drainage, and the full complexity of the ocular surface, all of which may influence drug transport and ocular exposure in vivo.

**Figure 7:**
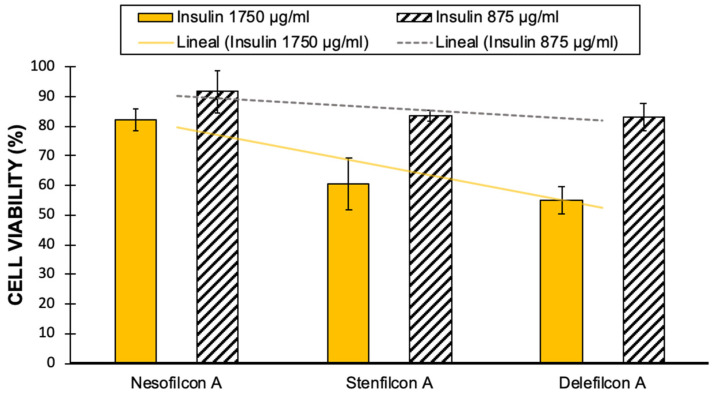
Cell viability (%) of HCE-2 cells measured by the MTT assay after 24 h exposure to insulin-loaded SCLs.

**Figure 8:**
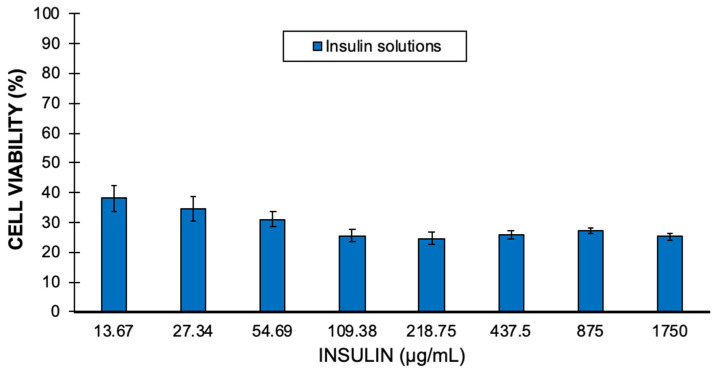
Cell viability (%) of HCE-2 cells measured by the MTT assay after 24 h exposure to free insulin solutions.

Although higher insulin concentrations may enhance ocular penetration, they may also increase the risk of ocular toxicity [97]. Based on these considerations, the corneal epithelial cell model was employed to evaluate the effects of SCL compositions on cell viability (*n* = 12) [98], together with the spatial separation approach described above. Higher cell viability was observed for Nesofilcon A at both concentrations compared with the other materials (Figure 7). Statistically significant differences were detected between the two insulin concentrations within the same material (*p* ≤ 0.006), with higher viability observed at the lower insulin concentration (875 µg/mL). The cytotoxicity of free insulin solutions (13.67–1750 µg/mL) resulted in a lower epithelial cell survival rate (<40%), whereas insulin-loaded SCLs (Figure 8) were associated with higher cell viability under the conditions tested (*p* < 0.001). These findings suggest a potential reduction in cytotoxic effects when insulin is incorporated into the SCL matrix. This may be related to a more gradual exposure to the drug when delivered from the lens matrix rather than as a free solution. According to ISO 10993-5:2009 [99], Stenfilcon A (60.70 ± 8.78%) and Delefilcon A (54.95 ± 4.67%) loaded with 1750 µg/mL insulin did not meet the minimum viability threshold, whereas the remaining formulations exhibited cell viability values above 80%, supporting their suitability as safe biomaterials for ocular drug delivery [100]. Based on these findings, a loading concentration of 875 µg/mL appears to be sufficient to achieve a therapeutic effect while maintaining a favorable safety profile.

The most relevant limitation of the present study was the use of 10 mm lens discs due to the size of the polyester membrane employed in the permeability studies, which may not fully replicate the behavior of intact lenses under physiological conditions. In particular, the reduced sample size may slightly affect drug distribution and release kinetics compared with full-sized lenses, mainly due to changes in surface-to-volume ratio and edge effects. Furthermore, during the cytotoxicity assay, the lenses remained in static contact with the culture medium for 24 h without tear renewal. This static condition may underestimate in vivo cell viability, as continuous tear renewal contributes to drug dilution and clearance in the ocular environment.

To the best of our knowledge, this work represents the first evaluation of commercially available SCLs loaded with insulin, highlighting their potential as a promising platform for ocular drug delivery and corneal therapeutic applications.

## 4. Conclusions

The conventional hydrogel (Nesofilcon A) and silicone hydrogel (Stenfilcon A and Delefilcon A) commercially available soft contact lenses evaluated in this study enabled sustained insulin release for at least 8 h and effective permeation across an in vitro corneal epithelial cell barrier over a 24 h period. Furthermore, cell viability assays demonstrated a markedly improved safety profile for insulin-loaded lenses compared with free insulin solutions, with cell viability values exceeding 80% for all materials loaded with 875 µg/mL insulin. This reduced cytotoxicity is likely associated with the role of the contact lens as a drug reservoir, which modulates drug exposure and prevents high local concentrations, thereby exerting a protective effect on epithelial cells and supporting overall biocompatibility.

From a clinical perspective, most of the evaluated materials preserved their physicochemical properties within ISO 18369-2:2017 tolerance limits following insulin loading, indicating that drug incorporation does not substantially compromise key optical properties. Overall, these findings suggest that insulin incorporation into commercially available soft contact lenses can achieve a balance between sustained drug delivery and ocular biocompatibility without significantly altering lens performance in most cases.

This is, to the best of our knowledge, the first study to investigate the in vitro loading and release of insulin from different commercial SCL materials using a human corneal epithelial cell model, together with a systematic safety evaluation. Further in vivo studies are warranted to confirm these findings and to support the translation of this platform into clinical applications for ocular drug delivery and corneal wound healing.

## Figures and Tables

**Table 1:** Technical specifications of the soft contact lenses used in the present study.

	Acuvue^®^ Moist	PureVision^®^	Biotrue^®^	Dailies Total1^TM^	Total30^TM^	MyDay^®^	Biofinity^®^	Air Optix^TM^ Night & Day^TM^
Manufacturer	Johnson & Johnson	Bausch & Lomb	Bausch & Lomb	Alcon	Alcon	CooperVision	CooperVision	Alcon
Material	Etafilcon A	Balafilcon A	Nesofilcon A	Delefilcon A	Lehfilcon A	Stenfilcon A	Comfilcon A	Lotrafilcon A
Group ISO 18369-1:2017 [24]	IV	Va	II	Vc	Vb	Vb	Vc	Vc
Ionic	Yes	Yes	No	No	No	No	No	No
Dk/t	25.5	112	42	156	154	100	160	175
EWC	58	36	78	33–80	55–≥90	54	48	24
RI	1.4	1.426	1.374	1.4267	1.4	1.401	1.4	1.43
CT (−3.00 D)	84	90	100	90	80	80	80	80
Base curve radius (mm)	8.5	8.6	8.6	8.5	8.4	8.4	8.6	8.4
Diameter (mm)	14.2	14.0	14.2	14.1	14.2	14.2	14.0	13.8

SCLs Groups ISO 18369-1:2017: conventional hydrogel (II, IV) and silicone hydrogel (V); Dk/t: oxygen transmissibility (×10^−9^[cm/s] [mLO_2_/mL∙mmHg]); EWC: equilibrium water content (%); RI: refractive index; CT: central thickness for a −3.00 D lens (μm).

**Table 2:** Stabilization time (ST, min) and valid dehydration (VD, %) for each contact lens material and dioptric power in the dehydration process.

Material	Power +6.00 D (*n* = 3)		Power −0.50 D (*n* = 3)		Power −6.00 D (*n* = 3)	
	ST (min)	VD (%)	ST (min)	VD (%)	ST (min)	VD (%)
Etafilcon A	30	97.62	30	97.94	35	97.43
Balafilcon A	20	97.40	20	97.00	20	97.40
Nesofilcon A	35	99.62	35	99.72	40	97.83
Delefilcon A	30	98.92	30	98.95	30	99.74
Lehfilcon A	30	99.64	30	99.02	35	98.48
Stenfilcon A	30	98.37	30	99.11	30	97.81
Comfilcon A	30	98.73	30	98.80	30	98.02
Lotrafilcon A	30	98.42	25	99.99	30	98.60

**Table 3:** Equilibrium water content (EWC, %) and wettability (TFSQ values) for all non-loaded contact lenses immersed in PBS (control), and for insulin-loaded (insulin) soft contact lens materials. Values are expressed as mean ± SD. * *p* < 0.05, Student’s *t*-test for related samples.

Material (*n* = 9)	EWC (%)			Wettability		
	Control	Insulin	*p*-Value	Control	Insulin	*p*-Value
Etafilcon A	53.17 ± 1.39	56.51 ± 0.50	0.001 *	0.710 ± 0.266	0.325 ± 0.135	0.002 *
Balafilcon A	35.32 ± 1.06	38.72 ± 2.45	0.001 *	0.820 ± 0.246	0.683 ± 0.243	0.291
Nesofilcon A	75.78 ± 0.59	75.50 ± 0.39	0.040 *	0.416 ± 0.127	0.514 ± 0.197	0.262
Delefilcon A	30.25 ± 1.11	32.10 ± 1.85	0.009 *	0.491 ± 0.178	0.420 ± 0.052	0.288
Lehfilcon A	53.47 ± 0.63	53.74 ± 0.80	0.247	0.450 ± 0.173	0.491 ± 0.108	0.597
Stenfilcon A	54.37 ± 0.79	54.96 ± 0.88	0.052	0.611 ± 0.207	0.585 ± 0.173	0.702
Comfilcon A	47.37 ± 0.66	47.23 ± 1.08	0.706	0.540 ± 0.161	0.514 ± 0.121	0.614
Lotrafilcon A	25.29 ± 0.87	26.50 ± 1.87	0.096	0.387 ± 0.173	0.735 ± 0.210	0.007 *

**Table 4:** Physical parameters (base curve radius and diameter) before (control) and after loading the SCLs with the insulin solution (insulin). Values are expressed as mean ± SD. * *p*-value < 0.05, Wilcoxon test for paired samples.

Material	Base Curve Radius (mm)			Diameter (mm)		
	Control	Insulin	*p*-Value	Control	Insulin	*p*-Value
Etafilcon A	8.51 ± 0.03	8.51 ± 0.03	1.00	14.14 ± 0.05	14.14 ± 0.05	1.00
Balafilcon A	8.59 ± 0.03	8.59 ± 0.03	1.00	14.00 ± 0.05	14.00 ± 0.05	1.00
Nesofilcon A	8.60 ± 0.00	8.60 ± 0.00	1.00	14.18 ± 0.04	14.18 ± 0.04	1.00
Delefilcon A	8.50 ± 0.05	8.47 ± 0.05	0.08	14.10 ± 0.00	14.11 ± 0.03	0.32
Lehfilcon A	8.41 ± 0.03	8.41 ± 0.03	1.00	14.19 ± 0.03	14.19 ± 0.03	1.00
Stenfilcon A	8.42 ± 0.04	8.41 ± 0.06	0.32	14.17 ± 0.05	14.14 ± 0.07	0.32
Comfilcon A	8.58 ± 0.04	8.58 ± 0.04	1.00	13.97 ± 0.07	13.97 ± 0.07	1.00
Lotrafilcon A	8.39 ± 0.06	8.39 ± 0.06	1.00	13.77 ± 0.05	13.77 ± 0.05	1.00

**Table 5:** Central thickness (µm) and refractive index of the different SCL materials and powers before (control) and after insulin loading. Values expressed as mean ± SD. * *p*-value < 0.05, Wilcoxon test for paired samples.

Material	Power (D)	Central Thickness (µm) (*n* = 3)			Refractive Index (*n* = 9)		
		Control (PBS)	Insulin	*p*-Value	Control (PBS)	Insulin	*p*-Value
Etafilcon A	+6.00	221.80 ± 3.97	222.13 ± 1.97	0.042 *	1.402 ± 0.001	1.402 ± 0.001	0.408
	−0.50	115.65 ± 2.27	118.60 ± 2.47				
	−6.00	71.75 ± 0.98	75.02 ± 1.14				
Balafilcon A	+6.00	187.52 ± 2.95	191.06 ± 2.56	0.236	1.425 ± 0.001	1.425 ± 0.001	0.750
	−0.50	87.49 ± 3.39	81.70 ± 1.47				
	−6.00	83.63 ± 7.37	80.09 ± 6.02				
Nesofilcon A	+6.00	200.62 ± 0.58	196.28 ± 2.65	0.293	1.373 ± 0.001	1.375 ± 0.001	0.016 *
	−0.50	95.81 ± 2.31	97.48 ± 3.52				
	−6.00	101.48 ± 4.17	115.83 ± 4.51				
Delefilcon A	+6.00	212.18 ± 9.50	198.04 ± 6.84	0.138	1.423 ± 0.002	1.425 ± 0.001	0.011 *
	−0.50	110.59 ± 2.01	105.13 ± 1.93				
	−6.00	87.77 ± 9.79	88.41 ± 1.48				
Lehfilcon A	+6.00	194.61 ± 9.38	201.16 ± 0.57	0.938	1.400 ± 0.001	1.401 ± 0.002	0.223
	−0.50	132.36 ± 4.85	129.08 ± 3.16				
	−6.00	88.46 ± 1.97	76.34 ± 1.50				
Stenfilcon A	+6.00	207.56 ± 9.28	215.09 ± 4.50	0.484	1.400 ± 0.001	1.401 ± 0.001	0.016 *
	−0.50	133.25 ± 5.67	131.61 ± 6.13				
	−6.00	85.77 ± 9.28	79.23 ± 1.14				
Comfilcon A	+6.00	254.89 ± 3.00	255.87 ± 5.41	0.343	1.406 ± 0.001	1.401 ± 0.002	0.007 *
	−0.50	124.50 ± 1.50	122.20 ± 5.04				
	−6.00	68.80 ± 0.98	64.54 ± 3.00				
Lotrafilcon A	+6.00	212.65 ± 2.89	215.54 ± 3.47	0.374	1.427 ± 0.001	1.428 ± 0.002	0.167
	−0.50	89.49 ± 3.34	85.00 ± 2.94				
	−6.00	74.41 ± 0.55	72.17 ± 1.93				

**Table 6:** Differences in refractive power (ΔD) between insulin-loaded and PBS-soaked (control) SCLs at distances of −6.0, −3.0, −0.5, +0.5, +3.0, and +6.0 mm from the center, as extracted from the power profiles. Mean values were calculated from three lenses per material and power.

Material	Power (D)	ΔD—SCLs Insulin vs. SCLs PBS (*n* = 3)					
		−6.0 mm	−3.0 mm	−0.5 mm	+0.5 mm	+3.0 mm	+6.0 mm
Etafilcon A	+6.00	−1.94	−0.17	−0.27	−0.27	−0.17	−3.70
	−0.50	−2.93	0.08	−0.04	−0.04	0.08	−4.99
	−6.00	−3.78	0.96	0.71	0.71	0.96	4.37
Balafilcon A	+6.00	3.79	1.64	0.64	0.64	1.64	3.79
	−0.50	4.52	0.05	0.11	0.11	0.05	4.52
	−6.00	2.73	−0.18	0.13	0.13	−0.18	2.73
Nesofilcon A	+6.00	−0.14	−0.01	0.00	0.00	−0.01	−0.14
	−0.50	−0.33	0.01	0.12	0.12	0.01	−0.33
	−6.00	−0.03	0.10	0.01	0.01	0.10	−0.03
Delefilcon A	+6.00	−0.27	0.44	0.35	0.35	0.44	−0.27
	−0.50	0.10	0.00	0.14	0.14	0.00	0.10
	−6.00	0.16	−0.09	0.15	0.15	−0.09	0.16
Lehfilcon A	+6.00	1.02	−0.12	−0.03	−0.01	−0.13	0.20
	−0.50	0.32	−0.02	0.44	0.44	−0.02	0.32
	−6.00	0.03	−0.11	−0.13	−0.24	−0.11	1.55
Stenfilcon A	+6.00	0.01	0.14	0.24	0.24	0.14	0.01
	−0.50	−0.37	−0.02	−0.21	−0.21	−0.02	−0.37
	−6.00	0.37	−0.13	−0.05	−0.05	−0.13	0.37
Comfilcon A	+6.00	−2.21	3.05	3.18	3.18	3.05	−2.21
	−0.50	−0.32	−0.06	−0.06	−0.06	−0.06	−0.32
	−6.00	−0.07	−0.25	−0.37	−0.37	−0.25	−0.07
Lotrafilcon A	+6.00	0.02	0.35	0.41	0.41	0.35	0.02
	−0.50	−0.09	−0.01	−0.05	−0.05	0.00	−0.25
	−6.00	−0.01	−0.18	−0.11	−0.11	−0.18	−0.01

**Table 7:** Insulin loaded and released into different SCL materials after soaking 10 mm discs in 1750 and 875 µg/mL insulin solutions.

Material (*n* = 6)	Unit	C_0_ = 1750 µg/mL		C_0_ = 875 µg/mL	
		Insulin Load	Insulin Release	Insulin Load	Insulin Release
Nesofilcon A	µg/mL	834.64 ± 2.32	58.81 ± 4.52	225.45 ± 30.77	33.69 ± 5.35
	%	47.69 ± 0.13	7.05 ± 0.53	25.77 ± 3.52	15.21 ± 3.27
Stenfilcon A	µg/mL	943.21 ± 24.81	150.59 ± 9.41	444.24 ± 17.86	74.21 ± 8.17
	%	53.90 ± 1.42	15.96 ± 0.87	50.77 ± 2.04	16.76 ± 2.25
Delefilcon A	µg/mL	934.87 ± 17.08	149.03 ± 8.12	417.52 ± 28.48	69.38 ± 7.86
	%	53.42 ± 0.98	15.96 ± 1.12	47.72 ± 3.26	16.78 ± 2.87

## Data Availability

The data used to support the findings of this study are available from the corresponding author upon request.

## Supplementary Materials

The following supporting information can be downloaded at: https://www.mdpi.com/article/10.3390/pharmaceutics18070779/s1, Figure S1: Spectra of insulin showing a maximum absorbance peak at 276 nm; Figure S2: Insulin calibration curve; Table S1: Technical specifications and polymer composition of the soft contact lenses used in drug delivery experiments; Figure S3: Estimated insulin loading (top) and insulin release (bottom), expressed as µg/mL.

## Abbreviations

The following abbreviations are used in this manuscript:

SCLs	Soft Contact Lenses
CH	Conventional Hydrogels
SH	Silicone Hydrogels
OCT	Optical Coherence Tomography
PBS	Phosphate-buffered saline
HBSS	Hanks Balanced Salt Solution
DR	Dehydration Rate
VD	Valid Dehydration
EWC	Equilibrium Water Content
RI	Refractive Index
OPL	Optical Path Length
MTT	Thiazolyl Blue Tetrazolium
