# Supplementary material for: In Vitro Characterization of Insulin-Loaded Soft Contact Lenses and Their Effect on Corneal Epithelial Cell Viability and Permeability
Source: Pharmaceutics. 2026 Jun 25;18(7):779. doi: 10.3390/pharmaceutics18070779 (PMC13415021; doi:10.3390/pharmaceutics18070779)
Supplement: Supplementary file 1 [file pharmaceutics-18-00779-s001.zip › pharmaceutics-4355855-supplementary.pdf]

Supplementary Materials

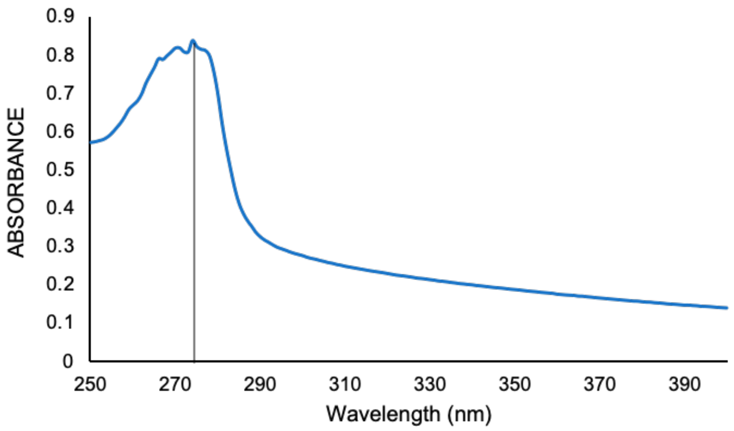

Supplementary Figure S1. Spectra of insulin showing a maximum absorbance peak at 276 nm.

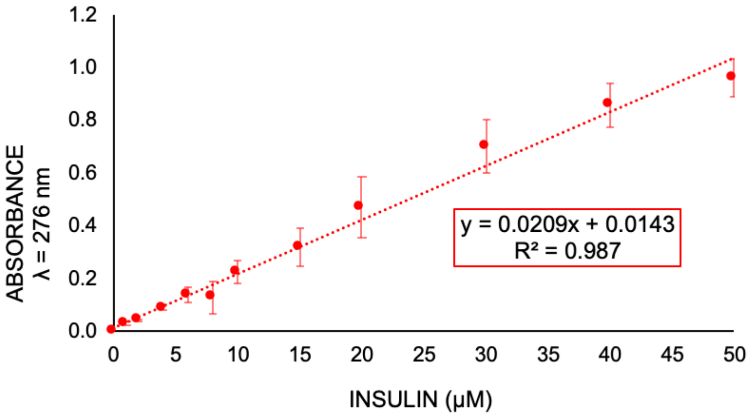

Supplementary Figure S2. Insulin calibration curve.

Supplementary Table S1. Technical specifications and polymer composition of the soft contact lenses used in drug delivery experiments.

	**Biotrue®**	**Dailies Total1™**	**MyDay®**
**Manufacturer**	Bausch & Lomb	Alcon	CooperVision
**Material**	Nesofilcon A	Delefilcon A	Stenfilcon A
**Group ISO 18369-1:2017 [24]**	II	Vc	Vb
**Polymer composition**	HEMA, PVP, NVP	Core: DMA, TRIS-AM, Si-MA Surface: PAMAM, AAA	EGDMA, EGMA, NB, PDMS, TEGDVE, VMA
**Ionic**	No	No	No
**Dk/t**	42	156	100
**EWC**	78	33 - 80	54
**RI**	1.374	1.4267	1.401
**CT (-3.00 D)**	100	90	80
**Base curve radius (mm)**	8.6	8.5	8.4
**Diameter (mm)**	14.2	14.1	14.2

SCLs Groups ISO 18369-1:2017: conventional hydrogel (II, IV) and silicone hydrogel (V); Dk/t: oxygen transmissibility ($x10^{-9}$[cm/s] [mLO_2/mL·mmHg]); EWC: equilibrium water content (%); RI: refractive index; CT: central thickness for a -3.00 D lens (μm).

Polymer composition—HEMA: poly-2-Hydroxyethyl methacrylate; PVP: Poly(vinylpyrrolidone); NVP: N-vinyl-2-pyrrolidone; DMA: N, N-Dimethylacrylamide; TRIS-AM: Tris(2-methacryloxyethyl) phosphate; Si-MA: Siloxane macromer; PAMAM: Polyamidoamine; AAA: N-allyl amino acetate; EGDMA: ethylene glycol dimethacrylate; EGMA: ethylene glycol methyl ether methacrylate; NB: norbloc, 2-[3-(2H-Benzotriazol-2-yl)-4-hydroxyphenyl]ethyl] methacrylate; PDMS: polydimethylsiloxane; PMMA: Poly methyl methacrylate; TEGDVE: triethylene glycol divinyl ether; VMA: N-vinyl-N-methylacetamide.

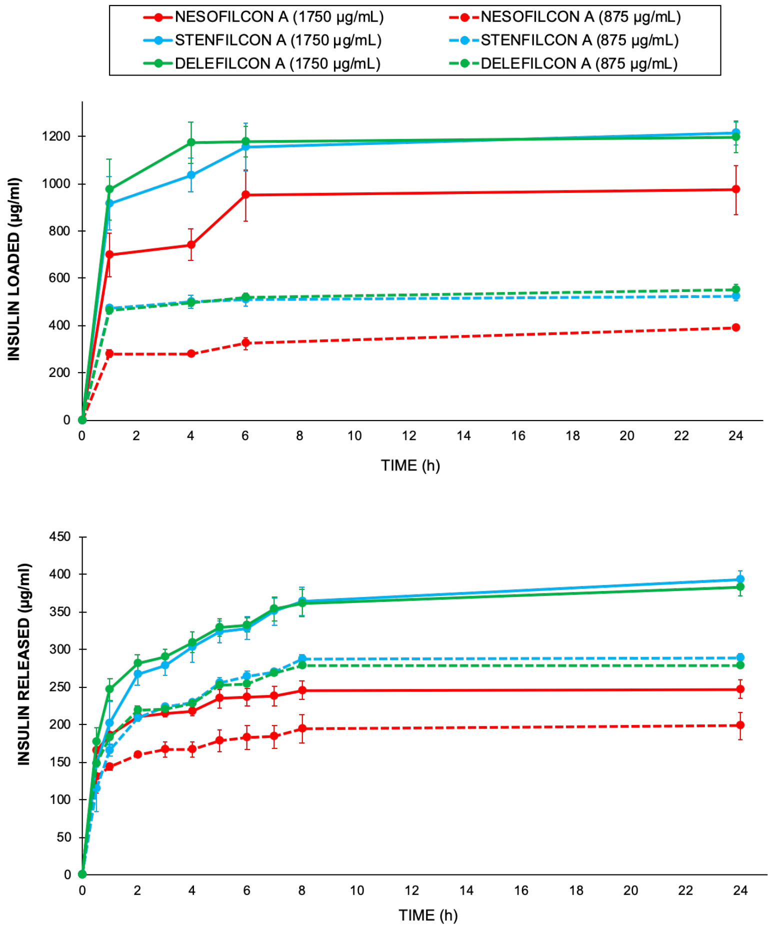

Supplementary Figure S3. Estimated insulin loading (top) and insulin release (bottom), expressed as µg/mL.
